# Immunosenescence and Vaccination

**DOI:** 10.1186/1742-4933-2-16

**Published:** 2005-11-24

**Authors:** Graham Pawelec

**Affiliations:** 1 Center for Medical Research (ZMF), University of Tubingen, Tubingen, Germany

## Abstract

The problems associated with the ageing immune system and vaccination were discussed recently at an international workshop at the Jenner Institute for Vaccine Research, Compton, UK, 6–7 October, 2005. This is a commentary on that session. The meeting included discussions on T and B cell differentiation and ageing, as well as dendritic cell and neutrophil data, with the emphasis on T cell immunosenescence, perceived as the most important hindrance to satisfactory responses to vaccines in the elderly. The main questions to be addressed in this context are the reasons for dysfunctionality of T cells in the elderly and what to do to improve T cell function. Several of the major reasons for poor T cell responses in the elderly were discussed; however, many important questions remain: The next meeting at the Jenner Institute may already be able to provide some of the answers to these questions, which have serious implications for public health issues in increasingly elderly populations.

## Introduction

An international workshop on Immunosenescence and Vaccination was organised at the Jenner Institute for Vaccine Research, Compton, UK, 6–7 October, 2005 by Peter Beverley (Jenner Institute), Arne Akbar (University College, London, UK) and Don Palmer (Royal Veterinary College, London, UK). The most profound clinical impact of age on the immune system concerns the response of the elderly to vaccination. The meeting included discussions on T and B cell differentiation and ageing, as well as dendritic cell and neutrophil data, with the emphasis on T cell immunosenescence, perceived as the most important hindrance to satisfactory responses to vaccines in the elderly. The main questions to be addressed in this context are the reasons for dysfunctionality of T cells in the elderly and what to do to improve T cell function.

## What is the problem?

Several of the major reasons for poor T cell responses in the elderly were discussed: is it a problem of stem cells with decreased potential for differentiation into naïve T cells? Is it a problem of the thymic environment not supporting this differentiation sufficiently any more? Is it a problem of naïve T cell ageing in the periphery and/or memory cell ageing and loss? The answers as they are emerging of course suggest that all these problems, and more, apply, as outlined below.

Diana Wallace and Peter Beverley [1] at the Jenner Institute, together with Derek Macallan in London, measured T cell turnover during human ageing by assessing the rate of uptake of deuterated glucose into DNA and its dilution with cell division. In healthy young people, CD45RO+ cells divide more rapidly than CD45RA+ cells in both the CD4 and CD8 subsets, whereby CD45RO+ cells have a 26 day half-life, with 2.7% of cells dividing every day, whereas the RA+ cells have a 154 day half-life and only 0.5% divide every day. Rather surprisingly, perhaps, this is the same in young and old, so these findings already suggest that memory T cells are turning over faster than naïve T cells. As the naïve cell division rate is the same in the elderly and the young, it is likely that naïve cells in the elderly have accumulated a larger number of population doublings during the life of the individual. This is consistent with and indeed explains the data on naïve cell ageing in mice from Laura Haynes [2] (Trudeau Institute, Saranac Lake, NY). However, it also suggests that if memory cell division is more rapid, replicative senescence of this population should occur faster. However, the rate of attrition of CD8+ CD45RA+ primed T cells was lower in the elderly than the young (by a factor > 10), and these persistent cells contained large clonal populations. This probably reflects acquisition of apoptosis-resistance by senescent CD8 cells and their accumulation in a dysfunctional state, as later described by Graham Pawelec [3] (Center for Medical Research, University of Tübingen, Germany). As these clonal expansions were to a great degree associated with seropositivity for Cytomegalovirus in the young, such data are consistent with many others presented here and elsewhere suggesting that CMV is a major driving force behind many of the measured manifestations of immunosenescence in humans. During this talk and others, there was much discussion on the impact of CMV on the human immune system and whether CMV in some way regulated (prevented) cell death in virus-specific, or even bystander, CD8 populations.

## Role of persistent antigenic stimulation

Chronic antigenic stimulation over a lifetime via a source resistant to elimination (persistent Herpes viruses, parasites, cancer, even autoantigens) may result in deleterious effects on the immune system. There is now a good body of evidence regarding the impact of CMV, and to a lesser extent, EBV infection on CD8 cells in young and elderly humans, but knowledge of its effects on CD4 cells is less well-developed, and on NK cells in the elderly almost lacking. However, Paul Moss [4] (Dept Cancer Studies, University of Birmingham, UK) reported preliminary studies showing little effect of CMV on NK cells, B cells or regulatory T cells in the elderly. He emphasised that CMV negatively influenced the number of naïve CD8 cells in the elderly and suggested that CMV accelerated age-associated processes which occur anyway, probably driven by other "subdominant" antigens. This is consistent with the much-discussed idea that chronic antigenic stimulation of any kind at any age can drive clonal expansions resulting in overall deleterious effects on immune function. Advanced age merely results in the accumulation of multiple such problems. Along these lines, evidence presented by Janko Nikolich-Zugich [5] (Oregon Health & Science Unversity, OR, USA) strongly suggests that even sustained proliferation not dependent on exogenous antigen (including that induced by lymphopenia or adjuvants) over a lifetime can induce persistent T cell clonal expansions in specific pathogen-free mice. In addition, he showed that persistent HSV-1 infection also led to the accumulation of clones specific for this virus, which also became dysregulated over time. These events were prevented by continuous antiviral treatment of infected mice, showing directly that subclinical herpesvirus reactivation is necessary to drive expansions of memory cells. G. Pawelec [3] pursued this line of thought by comparing chronic antigenic stimulation caused by persistent activating viruses in the elderly with that caused by tumour antigens in (mostly younger) cancer patients, as well as changes that can be observed in culture models in vitro. Functionally and phenotypically, many similarities in CD8 responses can be observed in the elderly and in cancer patients. The accumulation of CMV-specific CD8 cells bearing the hallmarks of anergic cells represents an important part of the "Immune Risk Phenotype" (IRP) predictive of mortality in the very elderly [6], and similarly dysfunctional cells in cancer patients may compromise the response to the tumour in an analogous fashion. The emergence of the IRP concept from the longitudinal ageing studies carried out by Anders Wikby and others in Sweden, and studied as a long-term collaborative project supported by the European Commission (currently under contract QLK6-CT-2002-02283, coordinated by G. Pawelec, see ) will hopefully also prove useful for assessing "biomarkers of immunosenescence" in younger individuals, and possibly cancer patients, as well. Recent work from the group has shown that CD8 clonal expansions driven predominantly by CMV (but also with EBV playing a part) begin already in early middle-age. We now know that remaining life expectancy in the very elderly (>85 yr) correlates with having greater numbers of different clonal expansions compared to those with few clonal expansions (but note that all individuals possess very large overall numbers of CMV-specific CD8 cells) [7]. We explain this by hypothesising that CMV drives clonal expansions of multiple CD8 cells recognising different CMV epitopes (in the young, up to 10% of the T cell repertoire is already obsessed with CMV, as shown by P. Moss [8] in his presentation). The number of different clones expanded increases with age, as does the overall number of CD8 cells recognising CMV epitopes. However, in very advanced age, this clonal diversity starts to shrink, as apoptosis-resistant senescent cells are more slowly lost by attrition (via a process not yet clarified) and individuals possessing only a small number of clonal expansions are now more at risk of death than those still retaining a larger repertoire. Why this is so, is also not yet clear, but it is felt unlikely that they die of CMV disease.

The presumably important role of CMV-specific CD4 cells in this context has not yet been assessed. In this direction, Arne Akbar [9] presented his studies on the functional status of CD4 cells in the elderly using assays which only reveal non-anergic cells, unlike the studies on CD8 cells, which revealed larger numbers of dysfunctional cells. This is because the production of equivalent MHC class II multimers is technically much more challenging than the class I reagents used for CD8 cells. Nonetheless, the data on those CMV-specific CD4 cells in the elderly which did remain functional were extremely informative. In seropositive donors, CMV-specific cells had shorter telomeres, a sign of increased proliferative history. CMV lysates stimulated secretion of type I interferon (IFN-α) from dendritic cells, which acted to inhibit telomerase function in all virus-specific cells (not just the CMV-specific cells) and to increase the fraction of CD27- CD28- cells. This may explain the unexpected finding that CTL in CMV-seropositives possess more differentiated phenotypes, whether they are CMV-specific or specific for unrelated antigens.

## What can be done to rejuvenate the elderly immune system?

Targeted neutralisation of CMV-stimulated IFN-α production might be beneficial in the CMV-positive elderly, according to the above-mentioned results. Other possibilities include the use of anti-viral agents or therapeutic vaccination, but neither of these approaches is likely to be available in the near future for use in the elderly. Nonetheless, agents such as valacyclovir have been developed, which have a good safety record in younger people and might be applicable to the elderly. Similarly, it seems unlikely that adoptive immunotherapy with ex-vivo-generated CMV-specific CD8 cells, despite being semi-routine in stem cell transplant recipients, would ever be easily transferable to the elderly. The elimination of the dysfunctional CMV-specific CD8 cells might be beneficial in removing potentially suppressive cells, or at the very least, simply making "space" for naïve cells that could in theory still be generated from residual thymic islands present in most elderly. How to target these, while leaving the functional CMV-specific cells intact, however, is a critical issue. Perhaps combining the use of KLRG-1 and CD57 to eliminate double positive cells might be a possibility, as proposed by G. Pawelec, because it is the small fraction of KLRG1+ but CD57- cells which seems to retain functionality in the elderly. One other possibility might be positive selection of the CMV-specific cells with a naïve phenotype (a very small number of which are present in most elderly, it seems) and expanding these ex vivo using IL 15. However, this would also require radical treatment involving ex vivo culture and adoptive immunotherapy, which might be practically difficult in the elderly. Along these lines, P. Beverley reported that naïve CD8 cells maintained in long-term culture in this way retained their naïve phenotype. These cultured cells upregulated telomerase and actually increased telomere length. This approach clearly also relies on the presence of naïve cells in the elderly, which could be isolated and expanded in culture. However, according to Beatrix Grubeck-Loebenstein [10] (Institute for Biology of Ageing, Innsbruck, Austria), there may not actually be any. She reported her search for truly naïve cells in the elderly, obtained by isolating CD8+ CD28+ CD45RA+ CD62L+ cells. Theoretically, these should be naïve, but she found that this subset also had shorter telomeres in the old than in the young, and, moreover, clonal spectratyping revealed a smaller repertoire in the former. So these cells have undergone considerable division, despite being phenotypially naïve. The implication is that even naïve cells in the elderly have "aged" in the same way as has been investigated in detail by L. Haynes [11] in the mouse model. She described that (in this case CD4+) naïve cells in old mice (CD28+, CD134+, CXCR5+) had low levels of CD40L expression (although this could be enhanced by IL 2 – use of which might also represent a potential avenue to remediation). When all the CD4 cells were depleted by antibody treatment, and the animals then left for 2 months to repopulate, new naïve cells did develop and even in old animals were now perfectly functional. These results do suggest that deleting accumulated dysfunctional cells, and allowing repopulation, can indeed "rejuvenate" the T cell system. Nonetheless, once again, it does not seem very likely that such radical approaches would be permitted in elderly people. However, focussing on the thymus and attempting to increase output of naïve cells in the elderly, whether or not combined with peripheral depletion, may still be a viable method for ameliorating immunosenescence. To this end, Richard Aspinall (Imperial College, London, UK) developed the strategy of targeting IL 7 [12] to the thymus only, to avoid cytokine side effects, by producing a fusion protein with CCR9, the receptors for which are expressed exclusively by thymic stromal cells. On challenge with influenza, mice had a decreased lung viral load when given the fusion protein, and fewer TNF-α-producing CD8 cells. The success of this approach requires not only that naïve cells are still present, but that in addition to the thymus, T cell progenitors are also still present and functional in the elderly. However, according to Ken Dorshkind [13] (UCLA, Los Angeles, USA), this may not be the case. He showed that "early T cell progenitors" (ETP; CD44+ CD25- c-kit^high ^CD127^-^) from old animals are fewer in number, more prone to apoptosis and have less proliferative capacity than in the young. Thus, if there is a block in the potential of hematopoietic stem cells to generate ETP, it would be difficult to see how IL 7 supplementation would succeed. One final possibility is the well-established approach of caloric restriction, discussed by J. Nikolich-Zugich [14] in the rhesus monkey. Calorically-restricted CMV+ monkeys have maintained numbers of naïve CD8 cells better than ad lib fed animals; they also had greater numbers of T cell receptor-excision circle-bearing cells (ie those with a lesser proliferative history) and lower levels of pro-inflammatory factors (TNF-α, IFN-γ). They also had fewer effector-memory cells. So perhaps even regarding the deleterious effects of CMV, the advice to eat less might still be some of the best available.

## Conclusion

Many questions remain. If CMV is really having such a disastrous effect in "accelerating" immunosenescence in the elderly, is it doing the same in the young? Are CMV-seronegative elderly donors healthier and do they live longer (and are centenarians CMV-seronegative?)? If CD4 and CD8 cells are equally affected, but B cells, dendritic cells and NK cells are really not (to be confirmed), are all types of the former equally affected (specifically, what about T-regulatory cells?)? Does CMV reactivate more frequently in the at-risk elderly, or does simply the duration of infection determine the level of immunosenescence? Do at-risk elderly ever die of disease which is related to CMV reactivation? Do CMV+ elderly people who have entered the IRP group ever leave it, and is this associated with survival benefit? Does it matter whether the source of chronic antigenic stress is CMV or can other antigens do the same? The next meeting at the Jenner Institute may already be able to provide some of the answers to these important questions which have serious implications for public health issues in increasingly elderly populations.
